# Deep Reinforcement Learning-Based Intelligent Security Forwarding Strategy for VANET

**DOI:** 10.3390/s23031204

**Published:** 2023-01-20

**Authors:** Boya Liu, Guoai Xu, Guosheng Xu, Chenyu Wang, Peiliang Zuo

**Affiliations:** 1 School of Cyberspace Security, Beijing University of Posts and Telecommunications, Beijing 100876, China; 2 Beijing Electronic Science and Technology Institute, Beijing 100070, China; 3 National Engineering Laboratory of Mobile Network Security, Beijing University of Posts and Telecommunications, Beijing 100876, China

**Keywords:** VANET, DRL, intelligent, message type, malicious node

## Abstract

The vehicular ad hoc network (VANET) constitutes a key technology for realizing intelligent transportation services. However, VANET is characterized by diverse message types, complex security attributes of communication nodes, and rapid network topology changes. In this case, how to ensure safe, efficient, convenient, and comfortable message services for users has become a challenge that should not be ignored. To improve the flexibility of routing matching multiple message types in VANET, this paper proposes a secure intelligent message forwarding strategy based on deep reinforcement learning (DRL). The key supporting elements of the model in the strategy are reasonably designed in combination with the scenario, and sufficient training of the model is carried out by deep Q networks (DQN). In the strategy, the state space is composed of the distance between candidate and destination nodes, the security attribute of candidate nodes and the type of message to be sent. The node can adaptively select the routing scheme according to the complex state space. Simulation and analysis show that the proposed strategy has the advantages of fast convergence, well generalization ability, high transmission security, and low network delay. The strategy has flexible and rich service patterns and provides flexible security for VANET message services.

## 1. Introduction

According to [1], 330 million cars have been connected to the Internet at the beginning of 2020, which form a huge VANET. The message forwarding strategy of the routing layer directly affects the data transmission performance of VANET. For a period of time, scholars have made in-depth research on VANET routing protocol and put forward a large number of routing strategies. Traditional routing strategies can be divided into location-based routing [2], cluster-based routing [3], broadcast routing [4], and multicast routing [5]. However, with the rapid development of the VANET, the message types generated and forwarded by nodes are constantly enriched. In the white paper “Web 5.0 Technology” [6], released in September 2021, the exchanged messages can be divided into ten deterministic levels. It is deduced that message types will continue to expand in the future, and traditional routing strategy might encounter more challenges. Meanwhile, the multi-hop transmission characteristics of VANET make it vulnerable to malicious node attacks. Therefore, routing strategy should also take security into account. In the face of the increasingly complex and diverse message transmission needs of users, it is necessary to design a routing strategy that the node can adaptively select the routing strategy, so as to reduce network overhead and improve the security and reliability of message forwarding in the VANET.

The matching selection of state–action pairs in reinforcement learning (RL) [7] makes it suitable for dynamic decision-making processes, which provides inspiration for solving routing problems. In particular, DRL combining RL and deep learning has been proposed in [8], which greatly increases the possibility of designing an intelligent security routing strategy. It can avoid the situation that the algorithm in RL sometimes cannot converge well in the face of complex state space, which further enhances the possibility of application of DRL in VANET. Scholars apply RL algorithms to VANET, which can optimize the quality of service (QoS) to multiple perspectives, such as delay, bandwidth, security, etc.

Several strategies have been proposed in recent years. Kumar et al. proposed a cluster-based routing protocol called the agent learning-based clustering algorithm (ALCA) [9]. Vehicle mobility is taken into consideration during the training of the agent to implement the clustering and routing mechanisms. In order to avoid route holes and reduce packet loss rate, X. Ji et al. [10] proposed an RHR algorithm in VANET based on RL technology. The algorithm does not rely on a single path, but on multipath exploration. At the same time, rewards are assigned to specific paths. The VRDRT algorithm was proposed in [11]. It relies on the roadside unit (RSU) to collect and maintain vehicle node information in a road section and uses DRL technology to predict the road section traffic situation in a period of time, and then selects the optimal next hop node. Y. Sun et al. [12] used the Q-learning algorithm to construct state space with the states of vehicle nodes in the network and performed greedy forwarding action to select vehicle nodes for data transmission with the reliability and stability of the path as parameters. With the help of UAV, B. S. Roh et al. [13] proposed Q-learning-based load balancing routing (Q-LBR). The algorithm defines data packets with high, medium, and low priorities. The UAV participated in node forwarding at the same time. X. Yang et al. [14] designed an algorithm that adopts a heuristic approach to accelerate the convergence speed of the Q-learning algorithm and selects forwarding nodes according to path reliability. A. Nahar et al. [15] proposed the RL-SDVN algorithm based on SDN and RL, which groups vehicles in the network and assists each other in finding the optimal path. L. Xiao et al. [16] proposed an RL-based anti-jamming VANET routing protocol (RAVR) with the help of a UAV and a jammer and achieved the purpose of taking correct actions to resist malicious nodes through continuous learning. An adaptive routing protocol based on RL was proposed by J. Wu et al. [17]. They designed a new Q value update function and used the data-forwarding mechanism and the MAC layer feedback mechanism. It effectively solves the routing loop, linkage interruption, and other issues. In order to improve the performance of data packet transmission and end-to-end delay, S. Jiang et al. [18] designed an auxiliary geographic routing based on Q-learning. A joint beacon rate and transmission power control based on Q-learning was proposed by J. Aznar-Poveda et al. [19] to ensure the timeliness of message forwarding. W. Jabbar et al. [20] studied the security characteristics of three different mobility models in the VANET. M. G et al. [21] proposed a trust evaluation scheme based on packet delivery rate and delay, which was adopted to identify spurious transmission and find the optimal path for message to reach the destination. An all-round and highly privacy-preserving location-based routing (ARPLR) scheme [22] relied on RSU to assist location management and used encryption to enhance privacy. Furthermore, a VANET location-based routing was proposed to ensure end-to-end secure communication between source and destination vehicles. Zhang et al. [23] proposed a DRL-aided trust-based VANET routing algorithm (TDRRL), which is designed for the SDN network paradigm. An intersection-based routing method using Q-learning (IRQ) [24] was presented for VANET by Khan. M.U et al. Based on the diversified types of messages to be transmitted, an adaptive routing method for the VANET message types based on reinforcement learning (RL) was proposed by Guoai Xu et al. [25]. Lansky. J et al. [26] proposed a categorization of RL-based routing schemes in VANET.

However, most of the current research work focuses on algorithm innovation and improvement under the premise of a fixed target, and generally lacks consideration of the security of VANET, while even less attention is paid to the adaptive selection of forwarding paths for diverse message types. This paper proposes an intelligent security message forwarding strategy for VANET based on DRL. The strategy takes the vehicle node delay, security attributes (trust score and whether it is a malicious node), distance from the destination node, message type, and other related parameters such as the state space to comprehensively build the state of the vehicle node. The DQN approach is used to select the optimal forwarding path, which has been verified to effectively improve the security and reliability of message forwarding in VANET, and also meet the requirements of efficient and convenient message service for vehicle nodes.

The remaining contents are arranged as follows: Section 1 introduces the preparatory knowledge related to the paper. Section 2 provides a complete description of the system model. Section 3 covers the algorithm modeling and algorithm process combined with DRL. In Section 4, the performance of the proposed method is verified. Section 6 summarizes the paper. In order to facilitate readers to better understand the proposed method, the main notations in the paper are listed in Table 1.

## 2. System Model

**Figure 1:**
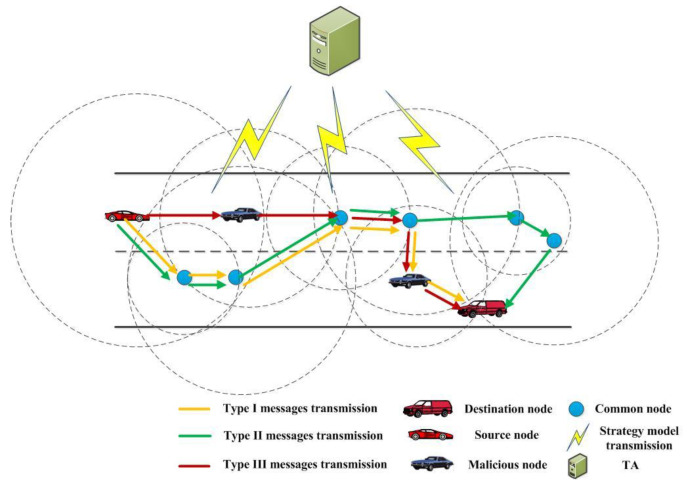
Schematic diagram of routing decision scenario.

Figure 1 shows the considered scenario of diversified message transmission with malicious nodes in the VANET. The model is mainly composed of vehicle nodes and trusted authority (TA) entities. Without the loss of generality, it is assumed that the vehicle node is equipped with an on-board unit, different types of sensors, and positioning and navigation systems to obtain the status of the surrounding vehicle nodes. Meanwhile, status data may include message delay, security attributes (i.e., trust score or an indication value denoting whether it is a malicious node), distance from destination node, message type, etc. Additionally, in order to illustrate the situation clearly, three types of messages are used in the figure to show the transmission process. Different vehicle nodes also have different security attributes and communication capabilities (such as signal transmission power and available bandwidth, etc.). It is worth noting that in the networking scenario, there may be multiple groups of source nodes and destination nodes as the vehicle moves and the time changes. The source node and destination node is not fixed and not limited. Moreover, different messages may have different requirements on transmission security, stability and timeliness.

The vehicle nodes in the model are divided into source nodes, destination nodes, common nodes and malicious nodes. The malicious nodes therein might have various malicious behaviors, which could be mainly divided into attacks against the validity of VANET communication, attacks against the authenticity and integrity of messages, and attacks against the confidentiality of information [27]. In our model, a malicious node pretends to be a normal node in the message forwarding process. After a message is transmitted to a malicious node, the malicious node forwards the message in violation of the preset routing strategy. Irregular forwarding of messages may affect the effective forwarding of messages, resulting in attacks on the validity of VANET communications. At the same time, it may also leak user information, so that illegal people benefit from it, resulting in confidentiality attacks against the message.

There are many different kinds of message types in VANET. The development and application of intelligent vehicle autonomous driving has spawned a variety of different message types, such as emergency brake warning for driving safety, traffic flow change warning, lane change warning, anti-collision warning and so on. In order to intuitively reflect the message types, we summarize and analyze the message types in VANET at the present stage. It can be inferred in [6] that the types of derived messages can be modeled by adjusting the urgency and reliability weights. For the convenience of expression, the weights of urgency and reliability are measured by high and low, respectively. We further divide message types into urgent messages with high reliability requirements (Type I messages), non-urgent messages with high reliability requirements (Type II messages), and urgent messages with low reliability requirements (Type III messages). In practice, the type and number of message types can be adjusted according to specific requirements. Among them, Type I messages refer to the information that can directly affect the driving safety of the vehicle, such as the driving state of the vehicle itself. This information requires high timeliness and reliability and should not be lost during the transmission process. Type II means the information may affect the driving safety of vehicles, such as congestion, traffic density, and other information. This kind of information is required to be accurately transmitted to vehicle nodes. Type III denotes the information can slightly affect the driving process of the vehicle, such as road surface information, road section information, etc. This information is required to be delivered to the vehicle node in real time.

Furthermore, it is assumed that the vehicle node can only communicate with the nodes within its communication range (the dotted circle in Figure 1) and obtain the states from a certain number of surrounding nodes. Meanwhile, the vehicle node will periodically send the obtained state data back to TA, which is responsible for training the model (which corresponding to the routing strategy) and sending the optimized model to each vehicle node for use. In particular, the trained model can be deployed on the vehicle node for a long time and only needs to be updated occasionally or regularly. Our strategy uses a distributed TA, either a traffic regulator or other designated regulator, for responding to VANET topology changes. Each TA is responsible for managing a domain, which can be adjusted as needed. In addition, according to the strategy model delivered by TA, the vehicle node selects the forwarding node according to the message type to be forwarded and the status of surrounding nodes. The forwarding node then selects the next hop node according to the current message type and the status of surrounding nodes obtained by itself. Finally, the forwarding nodes repeat the process until the message is transmitted to the destination node.

## 3. Preliminary Knowledge

### 3.1. Reinforcement Learning

RL is an important field of artificial intelligence. In order to make the agent get the maximum reward in the process of interaction with the environment, it denotes a kind of learning mapped from the state space S to the action space A. The goal of RL is to learn a strategy to maximize the expected reward [28]. Among the numerous algorithms of RL, Q-learning is one of the most representative ones, which can be described with the following instructions:

1. Execute the strategy and generate samples: $s,a,r,s^{\prime }$. where s denotes the state and the description of the environment; s∈S. a is the action reflecting the behavior of an agent; a∈A. r is the immediate reward value, that is, the reward fed back to the agent by the environment after the agent performs an action a according to the current state s. In addition, $s^{\prime }$ represents the next state after the environment is updated.
2. Estimate reward:
  (1) $$Q^{\pi }\left(s,a\right)\leftarrow Q^{\pi }\left(s,a\right)+\alpha \left[r+\gamma \max_{a^{\prime }}Q^{\pi }\left(s^{\prime },a^{\prime }\right)-Q^{\pi }\left(s,a\right)\right]$$
3. Where α represents the learning rate, γ is the attenuation value of the next state, and r denotes the immediate reward of the action a executed in the current state s. $\max_{a^{\prime }}Q^{\pi }\left(s^{\prime },a^{\prime }\right)$ represents the maximum Q value of the next state. Q-learning algorithm continuously executes and updates the value function $Q^{\pi }\left(s,a\right)$ to obtain the optimal strategy π.
4. Update strategy:
  (2) $$\pi \left(s\right)=\arg\max_{a\in A}Q\left(s,a\right),$$

### 3.2. Deep Reinforcement Learning

Q value cache may overflow in Q table when traditional RL faces large state space or action space. To solve this problem, DRL was proposed as an improved version of RL. Additionally, DRL uses RL to define problems and optimize goals, and adopts deep learning to solve modeling problems of policies and value functions. Then, it utilizes error backpropagation algorithms to optimize the objective functions. Based on the above advantages, DRL has general intelligence to solve complex problems to a certain extent and has achieved great success in many tasks.

In order to improve the stability of the algorithm and reduce the correlation between samples, the DQN algorithm mainly makes the following improvements to the traditional Q-learning algorithm:

1. The experience replay mechanism is used in the training process. The training samples are continuously accessed from memory units during training, and the stochastic gradient descent (SGD) algorithm is used to update parameters.
2. The current Q value in the DQN algorithm is approximated by a deep neural network (DNN), that is, $Q^{*}\left(s,a\right)=Q\left(s,a|\theta \right)$; θ represents the parameter of DNN. θ is updated in real time. The target value is generally approximated by $Y=r+\gamma \max_{a^{\prime }}Q\left(s^{\prime },a^{\prime }|\theta^{\prime }\right)$, and the network will copy the current network parameter to the target network parameter $\theta^{\prime }$ after every X rounds of iteration. The network parameter θ is updated by minimizing the mean square error between the current Q value and the target Q value, and the partial derivative of parameter θ can obtain the gradient:
  (3) $$L\left(\theta ,\theta^{\prime }\right)=E\left[{\left(Y-Q\left(s,a|\theta \right)\right)}^{2}\right],$$
  (4) $$\nabla_{\theta }L\left(\theta \right)=E\left[\left(Y-Q\left(s,a|\theta \right)\nabla_{\theta }Q\left(s,a|\theta \right)\right)\right],$$

## 4. Intelligent Message Forwarding Strategy

In the real scenario, the distance between each node and the destination node and its own security attribute may be different. In the same way, the message types may be different. Therefore, the strategy proposed in this paper can adaptively select a reasonable message forwarding path and transmit the message to the destination node. The details of the strategy, including state space, action space, and reward functions are shown in sequence.

### 4.1. State Space

We note that many status data of nodes may affect the performance of routing. To be specific, the delay of message transmission and the distance between the node and the destination node are more intuitive influencing factors. Different message types require node performance from the aspect of transmission timeliness. In particular, the trust score of a node and whether it is a malicious node will have a great impact on the transmission requirements of different message types. Therefore, the state space is defined as the state of each vehicle node at the moment, that is $state=\left\{t_{i,j},l_{j},d,\beta ,\lambda ,v\right\}$, where $t_{i,j}$ represents the delay of sending messages from the current node i to the surrounding node j. $l_{j}$ is the distance between the surrounding node j and the destination node. d is the representation of whether the current node is the destination node. If the d is the destination node, d=1, otherwise d=0. v is the representation of whether the current node is malicious node. If it is a malicious node, v=1, otherwise, v=0. β, λ represent the urgency and reliability of the current message type, respectively. This in turn determines the message types.

### 4.2. Action Space

The action space A can be described as the surrounding nodes of the vehicle nodes in the scene. It is modeled as: action={0,1⋯⋯,n}, where 0,1⋯⋯,n represents the surrounding nodes of the current node.

### 4.3. Reward Functions

Reward is used to reflect the quality level of the action made by the agent according to the environment. The assignment value of reward mainly refers to delay factor, distance factor, malicious node factor and message type. It should be noted that the reward is generally negative, and the maximum value of reward is 0 only when the selected next hop node is the destination node as follows:

If the next hop node is a malicious node:

(5) $$r=-\frac{1}{1+e^{-\left(\lambda -10\right)}}-\frac{1}{1+e^{-\left(\beta -10\right)}}\cdot \eta \cdot t_{i,j}-\left(1-\eta \right)\cdot l_{j},$$

where $t_{i,j}$ represents the time delay required for transmission from the current node i to node j, and $l_{j}$ denotes the distance between node j and the destination node. η is the weight factor that balances the weight of $t_{i,j}$ and $l_{j}$ in the reward function.

If the selected next hop node is not a malicious node, the strategy will judge whether the selected node is the destination node. Furthermore, if the destination node is selected, the maximum reward value will be obtained, r=0. Otherwise,

(6) $$r=-\frac{1}{1+e^{-\left(\beta -10\right)}}\cdot \eta \cdot t_{i,j}-\left(1-\eta \right)\cdot l_{j}$$

Note that in addition, if a “hit the wall” situation occurs (for example, the source node forwards the message to a direction where there is no vehicle node in the communication range in Figure 1), then r=1.

It can be seen from the analysis of Equations (5) and (6) that the next hop node is a malicious node, so the first term is added in (5), which is equivalent to the additional penalty term. In addition, β and λ can be assumed to be related to time and reliability, respectively. $\frac{1}{1+e^{-\left(\beta -10\right)}}$ and $\frac{1}{1+e^{-\left(\lambda -10\right)}}$ are typical sigmoid function $\frac{1}{1+e^{-x}}$. Its value ranges from 0 to 1. The characteristic is that when *x* = 0, the value is 0.5. In this paper, the demand of sending messages is characterized by reliability and urgency, and the strength of the demand of this nature is described by values of 1–30, respectively, where 1 represents very weak demand and 30 represents very strong demand. In Equations (5) and (6), when β and λ are equal to 30, $\frac{1}{1+e^{-\left(\beta -10\right)}}$ and $\frac{1}{1+e^{-\left(\lambda -10\right)}}$ are essentially equal to 1. When β and λ are equal to 1, $\frac{1}{1+e^{-\left(\beta -10\right)}}$ and $\frac{1}{1+e^{-\left(\lambda -10\right)}}$ are essentially equal to 0. This corresponds to the assignment logic of reward, that is, when the message urgent demand is strong, the value of β is very high. As well as the $\frac{1}{1+e^{-\left(\beta -10\right)}}$ being set to 1, the punishment corresponding to this item is heavier. The same is true when message reliability is high.

The algorithm process is shown in Algorithm 1. It should be noted that at the end of each training, the reward value of message forwarding path is recorded. If the total reward at this time is higher than the previous reward each time, it indicates that the forwarding strategy in the current training is optimal, and the network parameters at this time will be copied to the target network parameters. In other words, on the basis of the traditional DQN algorithm, the training algorithm can be improved continuously by updating the parameters of the target network.
**Algorithm 1.** Intelligent message forwarding (DRL-IMF) strategyInitialize: experience replay D, prediction network Q(s,a|θ) with network weight θ, target network $Q(s,a|\theta^{\prime })$ with network weight $\theta^{\prime }$, $\theta^{\prime }=\theta$, ε, $\varepsilon_{d}$ (decay rate of ε), $\varepsilon_{\min}$ (the minimal value of ε), reward route $P_{1}$, action route $P_{2}$, c=0, $r^{\prime }=0$.Input:source node and destination node, state S, action A, learning rate α, target network update frequency T.Output:route $P_{i,j}$.Step1:If $\varepsilon >\varepsilon_{\min}$, calculate $\varepsilon *\varepsilon_{d}$, and set $\varepsilon =\varepsilon *\varepsilon_{d}$.Step2: Generate a random number c, c∈(0,1).Step3:  If $0\leq c<\varepsilon_{m}$, select a from A.Step4:  else set $a=\arg\max_{a\in A}Q(s,a|\theta )$.Step5:  Save a and make state $s=s^{\prime }$, calculate r.Step6:  Save $\left(s,a,s^{\prime },r\right)$ into D, set $r^{\prime }=r^{\prime }+r$.Step7:  Select a batch of experience $D^{\prime }$ from D, calculate $L(\theta ,\theta^{\prime })$, update θ, then set c=c+1.Step8:  If nmodT=0, set $\theta =\theta^{\prime }$, and $s=s^{\prime }$.Step9:  If $s^{\prime }$ is the destination node, then save currently r.Step10:  If $r>r^{\max}$, then set $\theta =\theta^{\prime }$, and c=0.Step11:End

We now analyze the complexity of the proposed method. Assuming that the number of next hop nodes that can be selected by the current node is N, the complexity of the process of obtaining state information of the current node is O(12N). As the proposed method belongs to the offline type, its model has high complexity in the training process. However, the training of the model is not involved in the deployment and application process, so the analysis of its computational complexity does not include the training process. If a residual network containing M hidden layers is used as the DQN network of the method, and each layer has R neurons, then in the application process of the method, the complexity involved can be expressed as $O\left(\left(M-1\right)R^{2}+7NR\right)$ (under the condition of the worst network). Considering that M is generally small (for example, M=8 is set in the simulation in this paper), and N<R, the complexity of the proposed method can be expressed as $O\left(R^{2}\right)$. Similarly, we can calculate that the complexity of Q-LBR algorithm, which is adopted as a comparison method in the next section using the same network structure, is $O\left(\left(M-1\right)R^{2}+6NR+10N\right)$; then, its complexity can be simplified as $O\left(R^{2}\right)$.

## 5. Simulation and Analysis

### 5.1. Simulation Parameter Setting

In order to verify the effectiveness of the proposed method, this paper randomly generates multiple groups of VANET environments (topology snapshots of VANET) with malicious nodes. There are 48 vehicle nodes in each group of VANET environment, and 9 malicious nodes (non-source node and destination node) randomly in each group, and each group is trained with three message samples. Generally speaking, the message transmission of vehicle nodes has various demands. This paper (in the simulation) characterizes the message transmission demand with reliability and urgency. As mentioned, values 1–30 are used to describe the strength of the demand for this property, with 1 representing very weak demand and 30 representing very strong demand. In particular, only three message types are set in the simulation for performance verification in order to better render the method effect. β=30, λ=30 for Type I messages that are both urgent and reliable. β=1, λ=30 represents non-urgent but highly reliable Type II messages. β=30, λ=1 represents Type III messages that are urgent but have low reliability requirements. In practice, different message types can be characterized according to specific needs. The strategy in this paper is based on the DQN algorithm, which can also effectively deal with multiple complex message types.

In order to show the performance advantage of the proposed method, Q-LBR is selected as the comparison method in this paper. Considering that the scene targeted by Q-LBR is different from the scene concerned in this paper, Q-LBR cannot be directly applied to the scene in this paper. We made some modifications to the method. Therefore, we modify Q-LBR to some extent and reset the state to $state=\left\{t_{i,j},l_{j},d,\beta ,\mu_{j}\right\}$. Where $0\leq \mu_{j}\leq 1$ represents the normalized connectivity of node j. The higher the value, the more communication nodes around node j. The action space of Q-LBR is consistent with the method proposed in this paper, and its reward value is set as follows:

(7) $$r=\{\begin{array}{c} 0,\;if\;the\;destination\;node\;is\;selected\\ 1,\;if\;“hit\;the\;wall”\;situation\;occurs\\ -e^{\frac{1}{\mu_{j}}}-\frac{1}{1+e^{-\left(\beta -10\right)}}\cdot \eta \cdot t_{i,j}-\left(1-\eta \right)\cdot l_{j} \end{array}$$

As a preliminary study of intelligent message forwarding strategy, for the convenience of reference and without loss of generality, we assume that the vehicle node (source node) can obtain the actual status of at most n surrounding nodes through communication interaction, that is, one of n surrounding nodes can be selected for communication during routing selection. If the number of nodes is less than n, the part less than n can be filled. If there are more than n nodes, select n nodes with higher r values based on the surrounding nodes. This assumption does not affect the performance of the method and can meet the specific communication requirements at a certain time in the message forwarding of VANET, and also saves the message forwarding cost and storage space of the vehicle node to a large extent. In the simulation, n is set to 4.

The Keras platform is an open source artificial neural network library written in Python. It is often used to design, debug, evaluate, apply, and visualize deep learning models. In this paper, the Keras platform is used to train the deep Q network model of the proposed method. The size of the simulation area is set as 800 m × 800 m, where the spatial positions of vehicle nodes are randomly generated. It is worth noting that although this generation mode is slightly different from the actual road environment, this setting mode can show the performance of the proposed method and further ensure the generalization ability of the proposed method well. The packet size of all types of messages is set to 200 bytes, and the maximum communication distance of all nodes is set to 50 m. The parameters set by the simulation in this paper are summarized in Table 2. For each generated VANET environment (topology snapshots of VANET), the simulation trains it 500 times.

### 5.2. Convergence of the Method

**Figure 2:**
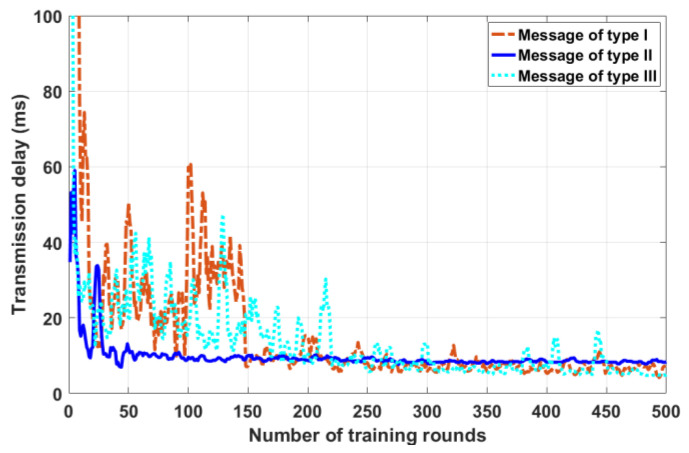
The proposed strategy is aimed at the convergence of random snapshots.

Firstly, the convergence of the proposed strategy is verified by simulation. Figure 2 shows the convergence of the proposed strategy for a randomly generated topological snapshot. As can be seen from the figure, the proposed strategy shows fast convergence characteristics for three different message types. Moreover, with the increase in the number of training rounds, the transmission delay of different messages in the proposed strategy shows a downward trend under the guidance of reasonable reward setting, and finally achieves convergence within 500 training rounds. This result preliminarily indicates the adaptability of the proposed strategy to the scenario of VANET. In order to verify the effectiveness of the proposed method, this paper randomly generates multiple groups of VANET environments (topology snapshots of VANET) with malicious nodes. There are 48 vehicle nodes in each group of VANET environment, and 9 malicious nodes (non-source node and destination node) randomly in each group. Each group is trained with three message samples. Generally speaking, the message transmission of vehicle nodes has various demands. This paper (in the simulation) characterizes the message transmission demand with reliability and urgency. As mentioned above, values 1–30 are used to describe the strength of the demand for this property, with 1 representing very weak demand and 30 representing very strong demand. In particular, only three message types are set in the simulation for performance verification to better render the method effect. β=30, λ=30 for Type I messages that are both urgent and reliable. β=1, λ=30 represents non-urgent but highly reliable Type II messages. β=30, λ=1 represents Type III messages that are urgent but have low reliability requirements. In practice, different message types can be characterized according to specific needs. The strategy in this paper is based on the DQN algorithm, which can also effectively deal with multiple complex message types.

### 5.3. Method to Compare the Details of Different Types of Messages

**Figure 3:**
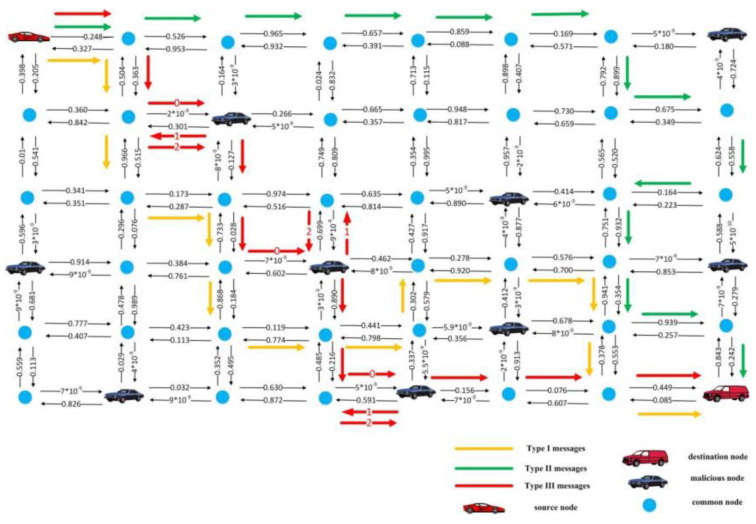
Schematic of the performance details of the proposed strategy for the three message types.

In order to more comprehensively show the processing details of the proposed strategy for different message types, we have a detailed representation of the solution’s message processing in Figure 3 (that is, the routing effect of the trained model for a topology snapshot). As can be seen from the figure, for the same network topology, although all three types of messages need to reach the same destination node from the same sending node, the routing process of the proposed schemes for the three types of messages is significantly different. For example, for Type I messages, data transmission needs to take into account reliability and timeliness. The proposed scheme in Figure 3 completely avoids malicious nodes to avoid inefficient data transmission. In contrast, for Type III messages, the transmission of messages has low requirements on reliability, so the routing of messages does not avoid being forwarded by malicious nodes, but mainly chooses the transmission path that can reach the destination node faster.

### 5.4. Performance for Random Snapshots

**Figure 4:**
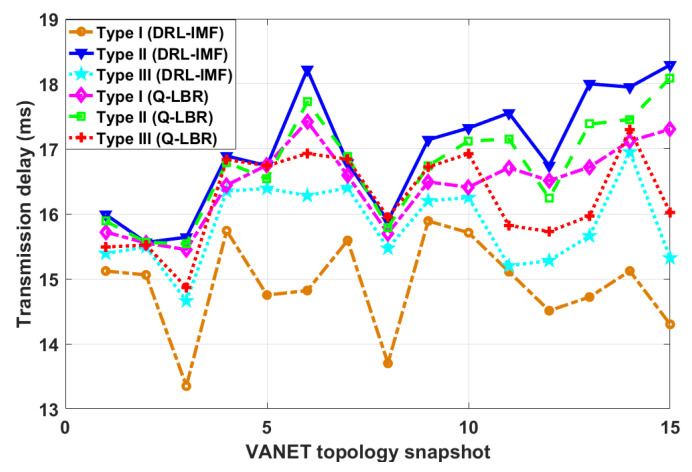
Performance comparison of the proposed strategy for three message types under random topology snapshot.

Then, the performance comparison of the proposed scheme against the three message types in the case of random topology snapshot is verified by simulation, and the results are shown in Figure 4. For the randomly generated topology snapshots, it can be seen that the transmission delays of the proposed schemes have obvious differences. The proposed strategy also shows different results for different message types. On the whole, the transmission delay of Type I messages is successively lower than Type III messages and Type II messages, which also matches the requirement intensity of the three message types for timeliness and reliability, indicating the effectiveness of the proposed strategy. For the comparison method Q-LBR, it shows different delay performance for three types of messages. Q-LBR has a lower delay value for Type I and Type III messages than for Type II messages, because it takes the timeliness requirement of messages into account in the setting of reward parameters. In addition, as can be seen from Figure 4, the delay performance of the Q-LBR method is only better than DRL-IMF for Type II messages on the whole. This is due to the fact that the Q-LBR only considers the reliability of the message. The above results reflect the adaptability of the proposed method to the message types and its performance advantages compared with existing methods.

### 5.5. Statistical Performance

The intelligent forwarding strategies are designed to optimize routing performance from different perspectives. By studying a large number of intelligent forwarding strategies, we compare the proposed strategies with several typical algorithms. As can be seen from Table 3, we mainly compare from several angles such as end-to-end delay consideration, vehicle’s position consideration, quick convergence mechanism, dependency on RSU, security feature, and message type consideration.

End-to-end delay optimization of a routing protocol ensures message delivery from a source node to a destination node in the minimum time. For fast and reliable message forwarding, the position of the vehicle needs to be considered. The reliance on RSU for message forwarding may limit the application of routing schemes. The fast convergence mechanism is a performance indicator that routes want to optimize. Security feature, as well as intelligent forwarding of multiple message types, are important factors in improving routing performance.

Among the methods in Table 3, the focus on optimizing performance varies. ACLA, TDRRL, and RAVR do not consider end-to-end optimization. ACLA, TDRRL, and Q-LBR do not take vehicle position into account. ALCA and TDRRL do not involve a fast convergence mechanism. Meanwhile, the four schemes compared with the scheme in this paper are all designed based on RSU, and the applicable scenarios have certain limitations. Our solution also has certain advantages in terms of security and flexible services.

Finally, the advantages of the proposed strategy are demonstrated from the perspective of performance statistics of the simulation results of 1000 random topology snapshots, where each snapshot is responsible for sending three different types of messages, as shown in Table 4. The DRL method in the table indicates that β and λ of the proposed DRL-IMF scheme are set to 15 and 5, respectively, to show the response effect of the scheme set for key parameters. We could clearly see from the results in the table that the proposed strategy has different delay and repeated forwarding node ratios for three different messages. Furthermore, since Type I and Type II messages require high reliability, the utilization rate of malicious nodes in the proposed strategy is 0, which indicates the matching between the efficiency of the proposed strategy and the potential demand of message types. In addition, it can be observed that the performance of greedy perimeter stateless routing (GPSR) is the worst due to the failure to consider the type of messages and other factors affecting the routing performance. The DRL approach is similar in performance to DRL-IMF (Type III). Specifically, DRL is slightly higher than DRL-IMF (Type III) in transmission delay and lower than DRL-IMF (Type III) in malicious node utilization. This is because the DRL method has an interleaved parameter setting with the DRL-IMF (type III). In addition, it can be seen from the table that the performance of Q-LBR method is different for different message types to some extent. Q-LBR does not consider the factor of malicious nodes, so the utilization rate of malicious nodes for the three types of messages are high. It is significantly different from the proposed method. For Type I and Type III messages, the average transmission delay of the Q-LBR method is lower than Type II messages. The reason is that both node connectivity and message timeliness are taken into account by the Q-LBR method. It can be easily observed from the table that the overall performance of Q-LBR is weaker than the DRL-IMF method. Therefore, it can be concluded from the table that the proposed strategy can well-adapt to and meet the requirements of different message types for transmission delay and reliability.

## 6. Conclusions

With the maturity of VANET applications, the types of messages gradually become rich. Aiming at the feature that different message types have different requirements for data transmission performance, this paper proposes an intelligent secure forwarding strategy based on DRL. We guide the efficient training of the DQN model by properly designing the state space and action space that match the VANET environment, and by relying on the matching reward settings. A large number of simulation results show that the proposed strategy could achieve fast convergence, meet the performance requirements of diverse message types. After complexity analysis and performance comparison, the comprehensive performance of the proposed method is better than the comparison method.

## Figures and Tables

**Table 1:** Main notations.

S	State space
A	Action space
$s,s^{\prime }$	Node state
a	Node action
r	Reward value
*α*	Learning rate
*γ*	The attenuation value of the next state
*π*	Optimal strategy
$\theta ,\theta^{\prime }$	DRL network parameter
X	Rounds of iteration
n	Number of node
$t_{i,j}$	The delay of sending messages from the current node *i* to the surrounding node *j*
$l_{j}$	The distance between the surrounding node *j* and the destination node
d	Indicates whether the node is a destination node
υ	Indicates whether the node is a malicious node
β,λ	The urgency and reliability of the message type
η	Weight factor in the reward function
ε	Decay rate
T	Target network update frequency
$P_{i,j}$	The route from node *i* to node *j*

**Table 2:** Simulation parameter settings.

Parameter	Values
Simulation area	800 m × 800 m
Number of vehicle nodes	48
Vehicular communication range	50 m
Message packet size	200 bytes
Vehicle moving speed	10~25 m/s
Number of message types	3
γ, ε, α, η	0.9, 0.6, 0.01, 0.7
The decay rate of ε	0.99
The minimum of ε is $\varepsilon_{\min}$	0.001
Experience playback memory bank capacity	300
Target network update frequency *T*	500
Experience playback mini-batch size	32
Training set/test set size	1000/300
Training times of a single snapshot	500
Number of surrounding candidate vehicle nodes n	4
Information transfer delay	0.1~2 ms (randomly generated)
Message type parameters β , λ	1 or 30

**Table 3:** Optimization performance comparison.

Method\Performance Parameters	ALCA	TDRRL	RAVR	Q-LBR	DRL-IMF
End-to-end delay consideration	×	×	×	√	√
Vehicle’s position consideration	×	×	√	×	√
Quick convergence mechanism	×	×	√	√	√
Dependency on RSU	√	√	√	√	×
Security feature	√	√	√	×	√
Message type consideration	×	×	×	√	√

**Table 4:** Performance statistics of the solution.

Method\Performance Parameters	DRL-IMF (Type I)	DRL-IMF (Type II)	DRL-IMF (Type III)	Q-LBR (Type I)	Q-LBR (Type II)	Q-LBR (Type III)	GPSR	DRL
Average time delay (ms)	10.28	14.21	11.86	12.76	14.01	13.15	15.47	12.05
Malicious node utilization rate	0%	0%	12.37%	11.29%	11.37%	11.85%	11.98%	11.55%
Ratio of repeat forwarding nodes	3.12%	3.09%	15.16%	13.45%	14.96%	13.56%	14.79%	14.58%

## Data Availability

The data presented in this study are available on request from the corresponding author. The data are not publicly available due to continue labeling the data. It may not be easy for readers to understand it now.
